# Effects of deep-sea water on training efficiency, locomotor function and respiratory metabolism in young and aged mice

**DOI:** 10.1016/j.heliyon.2024.e39296

**Published:** 2024-10-17

**Authors:** Koji Fukui, Riki Takeuchi, Yugo Kato, Nozomu Takeuchi, Hirotsugu Takenaka, Masahiro Kohno

**Affiliations:** a Molecular Cell Biology Laboratory, Department of Bioscience and Engineering, College of System Engineering and Science, Shibaura Institute of Technology, Fukasaku 307, Minuma-ku, Saitama, 337-8570, Japan; b Dydo-Takenaka Beverage Co., Ltd., Haneyou Ko 1310-1, Muroto, 781-6741, Japan

**Keywords:** Deep-sea water, Minerals, Exercise, Respiratory metabolism, Locomotor function

## Abstract

Deep sea water (DSW) contains many trace minerals, and its applications, which include its use as drinking water, have gradually been expanding. Generally, humans tend to be lacking in mineral intake and deficiencies of trace minerals may increase the risk of several health problems. In recent years, the lack of exercise among the elderly has become an issue, leading to the onset of frailty and sarcopenia, which in turn increases the risk of dementia. Therefore, we investigated whether the daily intake of DSW-extract-added water (DSW; hardness 300 mg/L) impacted the training effect in aged mice. Treatment with DSW significantly induced a training effect in aged mice subjected to treadmill exercise. Locomotor function and metabolic capacity were also significantly increased in aged mice after DSW treatment. The results indicate that daily intake of DSW may enhance the training effect of exercise and affect locomotor performance.

## Highlights

• Deep ocean water extract was added to enhance the training effect on elderly mice.
• Water supplemented with deep sea water extract affects locomotor activity and respiratory system in elderly mice.
• Magnesium and calcium may be important for this effect.
• Our results indicate that mineral intake not only compensates for mineral deficiencies but may also be effective against various age-related functional declines.

## Abbreviations

DSW: Deep sea water
RQ: Respiratory quotient
RER: Respiratory exchange ratio
BDNF: Brain-derived neurotrophic factor
NGF: Nerve growth factor
TrkA: Tropomyosin receptor kinase A
TrkB: Tropomyosin receptor kinase B
TP: Total protein
Alb: Albumin
BUN: Blood urea nitrogen
CRE: Creatinine
Na: Sodium
K: Potassium
Cl: Chlorine
Ca: Calcium
IP: Inorganic phosphorus
AST: Aspartate aminotransferase
ALT: Alanine aminotransferase
LDH: Lactic acid dehydrogenase
AMY: Amylase
T-CHO: Total cholesterol
TG: Triglyceride
HDL: High-density lipoprotein
T-BIL: Total bile acid
Glu: Glucose

## 1 Introduction

In addition to the four major essential elements of carbon, hydrogen, oxygen, and nitrogen, the human body is composed of 4 % minerals. Further, all minerals play an important role in sustaining life, even in small amounts. Minerals are divided into major and trace minerals; the former include sodium, potassium, calcium, magnesium, phosphorus, sulfur, and chloride, and the latter include iron, zinc, copper, manganese, selenium, chromium, molybdenum, cobalt, and iodine [1]. In Japan, the Ministry of Health, Labour and Welfare has established recommended daily intake amounts of major and trace minerals by age [2]. However, most age groups do not consume sufficient amounts of minerals on a daily basis [3,4]. There are two possible ways to prevent mineral deficiencies; one is to obtain sufficient minerals from the diet, and the other is to avoid the over-secretion of minerals from the body. However, it is difficult to reduce systemic mineral consumption even when the daily intake of minerals is not sufficient. In addition, stress is a major factor in modern life as a result of various lifestyle changes. It is considered that excessive stress induces oxidative stress, and that the intake of high levels of minerals such as zinc [5], iron [6], and selenium [7] has an antioxidant function. Accumulation of oxidative damage may increase the risk of developing several serious diseases such as cancers, arteriosclerosis, and neurodegenerative disorders, highlighting the importance of sufficient mineral intake [8,9].

To compensate for mineral deficiencies, mineral intake via supplements, fresh vegetables, and fruits is important. While mineral water is commonly used as a mineral supplement, we focus on deep-sea water (DSW) extract-added water (hereafter DSW) in this study. DSW, which is collected from a depth of at least 200 m, is used in various fields such as cosmetics and salt production [10]. DSW is characterized by high purity and low temperature-the surface water temperature is usually about 15–30 °C, but the temperature of DSW at the intake depth is about 10 °C throughout the year-as well as high mineral content of potassium, calcium, and magnesium [11,12]. The mineral content of drinking water is usually expressed in terms of hardness, and water hardness is calculated according to the amounts of calcium and magnesium [13]. Previously, we investigated the effects of different DSW hardness levels in obese mice. The treatment of obese mice with a water hardness of 300 mg/L showed greater beneficial effects in some behavioral tests compared to DSW with a hardness of 500 mg/L [14]. However, compared to the untreated group, serum sodium levels did not increase in the 300 mg/L-treatment group. Although, the results indicated that the continuous intake of DSW may have beneficial health effects, the detailed mechanisms underlying these beneficial effects of DSW intake remain to be elucidated. Unlike natural mineral water from Europe or America, most commercially available Japanese natural mineral water has a hardness of less than 100 mg/L. DSW may be more effective than natural mineral water collected from the mountains for mineral supplementation by drinking water.

In addition to regular beverages, most people drink mineral-rich “isotonic” drinks during and after exercise [15]. Isotonic drinks are said to be effective in relieving fatigue, and marathon runners drink them during races. Isotonic beverages contain various components, including amino acids, vitamins, and minerals [16]. However, the problem with isotonic drinks is that they ainly contain sodium as well as a lot of sugar. Consequently, there is a demand for beverages that allow people to consume a wide variety of minerals on a daily basis while avoiding excessive sugar intake.

In recent years, frailty and sarcopenia in the elderly have become a societal issue [17]. The increasing number of such patients may lead to an increase in patients with dementia in which the lack of social interaction and exercise have been shown to play a role. One of the causes of frailty and sarcopenia is suggested to be muscle weakness. Therefore, we proposed that mineral intake might be effective for mitigating muscle fatigue and weakness. In this study, to clarify the beneficial effects of DSW, water containing a DSW extract with a hardness of 300 mg/L was administered to young and aged mice, and the effects on endurance before and after treadmill training, locomotor and metabolic capacity, protein expression, and various serum indices were assessed.

## 2 Materials and Methods

### 2.1 Animals

The experiment was approved by the Animal Protection and Ethics Committee of Shibaura Institute of Technology (approval number #20003, approved on August 25, 2020). This approval complies with the Act on Welfare and Management of Animals in Japan. Male C57BL/6J mice, 1 and 20 months old, were purchased from Sankyo Labo Service Corp. Inc. (Tokyo, Japan) and Jaxon Laboratories Japan, Inc. (Yokohama, Japan). Each mice cohort was randomly assigned to two groups and provided either DSW extract-added water (hardness 300 mg/L) or control water to give the following group: Young (n = 15), Young + DSW (n = 15), Aged (n = 15), and Aged + DSW (n = 15). The DSW was produced by a joint research partner, Dydo-Takenaka Beverage Co., Ltd. (Kochi, Japan). All of the mice were given free access to a control diet (Labo MR Stock, Nosan Corp., Kanagawa, Japan) and drinking water. The number of animals in each experiment is described in the legend of each figure.

**Fig. 1:**
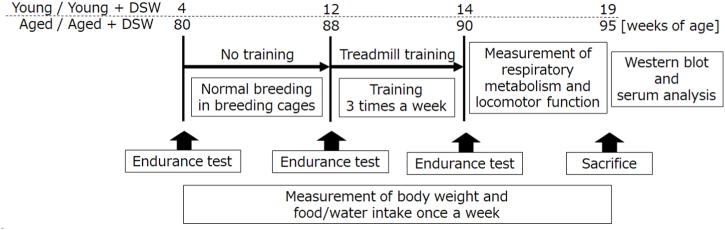
Experimental design.

Mice were provided the experimental and control water throughout the experimental period, i.e., up to 14 and 90 weeks of age, respectively (Fig. 1). Animals were housed at a temperature of 24 ± 2 °C under a 12-h light/dark cycle. Food and water intake and body weight were measured weekly using metabolic cages. Following the two-month feeding period, tests of behavior, respiratory metabolism, and locomotor function were conducted. The mice were anesthetized and immediately euthanized by decapitation; the cerebral cortex and serum samples were collected. All common chemical reagents were purchased from either FUJIFILM Wako Pure Chemical Industries, Ltd. (Osaka, Japan).

### 2.2 Water preparation

The DSW extract-added water provided by Dydo-Takenaka Beverage Co., Ltd. DSW was collected from the Muroto Deep-Sea Water Aqua Farm operated by Muroto City (Kochi, Japan) offshore of Cape Muroto in Kochi Prefecture (collection depth: ∼350 m, length of pipe used for water intake: ∼3000 m) [18]. The extract was concentrated by filtration (specific details are unknown due to the method being a trade secret), and the same extract was used to make the DSW extract-added water (hardness 300 mg/L). For the experimental treatment, the extract was diluted with filtered tap water. For the control treatment, filtered tap water was administered to the age-matched groups. An analysis of the dissolved elements in both water samples have been published previously [14].

### 2.3 Treadmill exercise experiment

#### 2.3.1 Endurance test before and after DSW treatment

The endurance of each mouse was assessed using a treadmill apparatus (#MK-690, Muromachi Kikai Co., Ltd., Tokyo, Japan). Before DSW administration, mice were allowed to roam freely for 10 min without belt activation to acclimate them to the apparatus. Next, mice were subjected to a running test in which the belt was accelerated from 1 m/min to 10 m/min over 20 min at an inclination angle of 0° (i.e., 1 m/min acceleration every 2 min). After a 10-min break, the angle of inclination was set to 10°, and the speed was gradually increased from 10 m/min to 30 m/min (in increments of 2 m/min every 4 min). The mice then ran at the same belt speed until they touched the shock grid for 5 s. The time and running distance were then recorded. In this experiment, mice were typically housed for 2 months before running training.

#### 2.3.2 Determining the strengthening effect of treadmill training by administering DSW

After 2 months of water administration and completion of the endurance tests, the mice were divided equally into two groups based on running distance. The training group ran for 60 min three times a week at a 0° incline. The running speed was 15 m/min in the first week and 18 m/min in the second week. Another group was housed normally without training in each cage for the same period as the first group.

### 2.4 Respiratory quotient and locomotor function

The respiratory quotient (RQ) is the ratio of carbon dioxide produced to oxygen consumed (VCO_2_/VO_2_) and was measured directly using a respiratory quotient measuring device (#MFD-RQ, SHINFACTORY, Fukuoka, Japan). This device is equipped with a locomotor capacity measuring device, a rotating cage, and a food intake measuring function. Mice were placed individually in the respiratory quotient measuring device, and were assessed for 24 h after 2 days of acclimatization.

#### 2.4.1 Locomotor function

The locomotor function of each mouse was measured using an infrared sensor device (ACTIMO-100RQ, SHINFACTORY). The number of times a mouse triggered the sensor in the metabolic cage was automatically counted and taken to reflect locomotor function.

#### 2.4.2 Respiratory quotient

The respiratory exchange ratio (RER) was calculated by dividing the VCO_2_ value by the VO_2_ value at the same time. For respiratory quotient measurements, VO_2_ and VCO_2_ were both automatically recorded at10-min intervals.

#### 2.4.3 Rotating score

Rotation of the cage was measured using an optional device (ROC-100, SHINFACTORY). Rotation speed (m/min) and time were recorded automatically at 10-min intervals.

### 2.5 Western blotting

After the treadmill assessment, as well as the respiratory metabolism and locomotor and locomotor function experiments, mice were sacrificed and the brain cortex region was harvested and homogenized in radio-immunoprecipitation assay buffer to obtain samples for Western blot analysis, as described previously [19]. A part of the dissected brain was homogenized at 30 vibrations per sec for 2 min using a TissueLyser II (QIAGEN K.K., Tokyo, Japan). After centrifugation at 15,000×*g* for 20 min at 4 °C, the protein concentration was measured (Bio-Rad protein assay, #500-0006JA, Bio-Rad Laboratories, Inc., Hercules, CA, USA). A total of 20 μg of each protein extract was then applied to, and separated on, a 12 % SDS-polyacrylamide gel before being transferred to a nitrocellulose membrane (ClearTrans cellulose nitrate membranes, 0.2 μm, FUJIFILM Wako Pure Chemical Co., Ltd.). A 2 % skim milk solution diluted with Tris-HCl-buffered saline (pH 7.6, 0.1 % Tween 20 (TBS-T)) was used as the blocking solution, and the transferred membrane was incubated for at least 1 h at room temperature. The transferred membrane was reacted with each primary antibody (anti-brain-derived neurotrophic factor (BDNF) (N-20) rabbit polyclonal antibody, 1:2500, #ab-108319 Abcam, Cambridge, UK; anti-nerve growth factor (NGF) (H-20) rabbit polyclonal antibody, 1:4000, #sc-548, SANTA CRUZ BIOTECHNOLOGY Inc., Dallas, TX, USA; anti-tropomyosin receptor kinase A (TrkA) (763), rabbit polyclonal antibody, 1:2000, #sc-118, SCBT Inc.; anti-tropomyosin receptor kinase B (TrkB) (H-181) rabbit polyclonal antibody, 1:7500, #sc-8316, SCBT Inc.) overnight at 4 °C. Anti-rabbit IgG horseradish peroxidase-conjugated antibody (Promega Corp. Madison, WI, USA) was used as the secondary antibody at 1:4000 dilution for over 1 h at room temperature. All signals were generated by incubation with chemiluminescent reagent (Immobilon; Merck KGaA, Darmstadt, Germany). For each protein band normalization, the transferred membranes were visualized with Ponceau S solution (#24-3875-5, Merck KGaA), and the relative expression ratio for each protein was determined using an LAS-3000 bioimaging system (FUJIFILM Corp., Tokyo, Japan). Expression ratios were calculated by dividing the value of each protein except BDNF and TrkB by the Ponceau S using ImageQuant TL software (ver.8.1, Global Life Sciences Technologies Japan, Tokyo, Japan).

### 2.6 Serum indices

Total protein (TP), albumin (ALB), blood urea nitrogen (BUN), creatinine (CRE), sodium (Na), potassium (K), chlorine (Cl), calcium (Ca), inorganic phosphorus (IP), aspartate aminotransferase (AST), alanine aminotransferase (ALT), lactic acid dehydrogenase (LDH), amylase (AMY), total cholesterol (T-CHO), triglyceride (TG), high density lipoprotein (HDL), total bile acid (T-BIL), and glucose (Glu) were measured by an external vendor (Oriental Yeast Co., Ltd., Tokyo, Japan).

### 2.7 Statistical analysis

All data are expressed as means ± standard error (SE) and GraphPad Prism software (ver.9.2.0, GraphPad Software LLC, San Diego, CA, USA) was used for statistical analysis. p-values of less than 0.05 were considered statistically significant. Details of the statistical methods are provided in each figure legend.

## 3 Results

### 3.1 Effect of DSW treatment on the body weight of young and aged mice

**Fig. 2:**
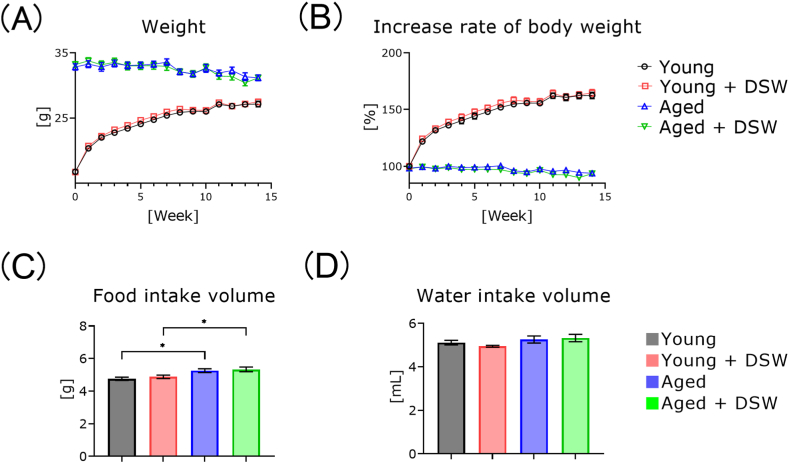
Effects of DSW treatment on body weight and food intake in young and aged mice. Average weight of each mouse group (A). Change in average weight of each mouse group over time (B). Food and water intake of each mouse group (C, D). The groups consisted of Young (n = 15), Young + DSW (n = 15), Aged (n = 15), and Aged + DSW (n = 15). Data are means ± SE. Data were analyzed using a two-way analysis of variance (A, B) and. a one-way analysis of variance followed by the Tukey-Kramer test (C, D). ∗p < 0.05.

The young mice gradually increased in body weight in a time-dependent manner with or without DSW treatment (Fig. 2A and B). The bodyweights of both age groups did not change during the treatment period with or without DSW treatment. Compared to the age-matched controls, the body weights of young and aged DSW-treated mice were not significantly different. The food intake of aged mice was significantly higher than that of the young mice (Fig. 2C). There were no significant differences in water intake among any of the groups (Fig. 2D).

### 3.2 Effect of DSW treatment on running distance in young and aged mice

**Fig. 3:**
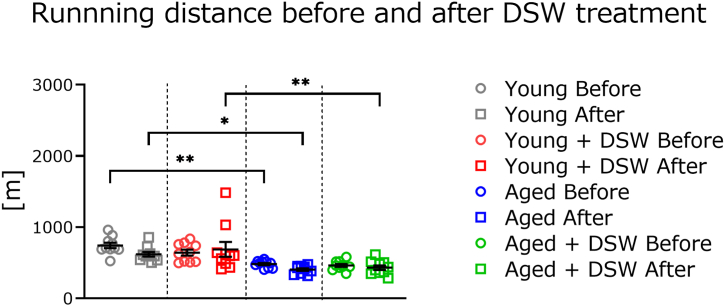
Difference in treadmill running distance of mice before and after 2 months of DSW treatment. All colored circles indicate the running distance before drinking DSW or filtered tap water. Squares indicate the running distance 2 months after treatment with each drinking water. The groups of consisted of Young (n = 10), Young + DSW (n = 10), Aged (n = 10), and Aged + DSW (n = 10). The data are shown as means ± SE. Data were analyzed using a one-way analysis of variance followed by the Tukey-Kramer test. ∗*p* < 0.05. ∗∗*p* < 0.01.

The running distance was measured before and after drinking DSW and filtered tap water, and there was no difference between the two groups. The results showed that endurance ability did not differ among each age group of mice. However, the running distance before start of the mineral supplementation experiment in the aged control group was significantly lower than that of the young control group (Fig. 3, gray and blue circles). Two months after treatment with each type of drinking water, the running distance of the young mouse groups was significantly higher than that of the aged groups with or without DSW treatment (gray vs blue squares, red vs green squares). There were no significant differences in running distance with or without DSW treatment in each mouse age group (gray vs red squares, blue vs green squares).

### 3.3 Effect of DSW treatment on the training effect of exercise in young and aged mice

**Fig. 4:**
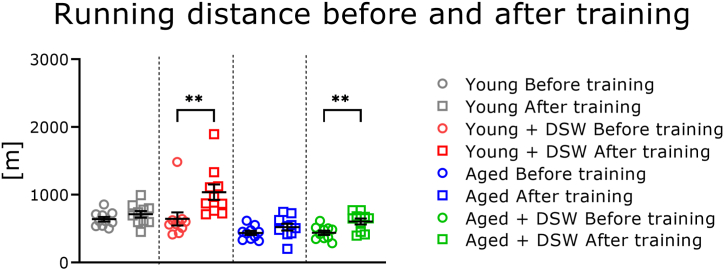
Treadmill running distance of young and aged mice treated with DSW before and after 2 weeks of training. Details of the training conditions are described in the Materials and Methods. The groups consisted of Young (n = 10), Young + DSW (n = 10), Aged (n = 10), and Aged + DSW (n = 10). The data are shown as means ± SE. Data were analyzed using a one-way analysis of variance followed by the Tukey-Kramer test. ∗∗*p* < 0.01.

After 2 weeks training, all mice were again subjected to the endurance test and the running distance was measured. Young and aged mice treated with DSW showed significantly increased running distance compared to that before the start of DSW-treatment (Fig. 4). These results indicate that long-term DSW treatment increased exercise endurance in mice.

### 3.4 Effect of DSW treatment on locomotor function in young and aged mice

**Fig. 5:**
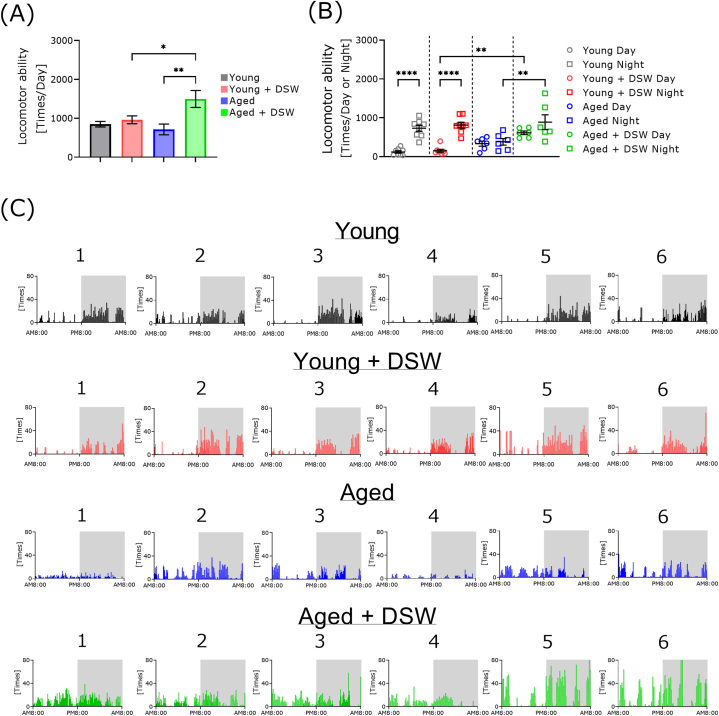
Effect of DSW treatment on the locomotor function of young and aged mice. Each mouse was placed in a respiratory quotient measuring device, and total locomotor function was measured for 24 h. The total locomotor function of each mouse was determined by the total number of crossings over 24 h captured by a reticulated laser in a sealed chamber (A). Locomotor functions of mice were segregated according to day and night (B). Locomotor functions of individual mice are shown (C). The groups consisted of Young (n = 8), Young + DSW (n = 8), Aged (n = 6), and Aged + DSW (n = 6). The data are shown as means ± SE. Data were analyzed using a one-way analysis of variance followed by a Tukey-Kramer test (A, B). ∗*p* < 0.05. ∗∗*p* < 0.01. ∗∗∗∗*p* < 0.0001.

Treatment with DSW significantly increased the daily locomotor function in aged mice compared to the age-matched control (Fig. 5A). Surprisingly, the total daily locomotor function of the aged DSW-treated mice was significantly higher than that of the young DSW-treated mice. Since mice are nocturnal, both young groups showed significantly increased locomotor function at night compared to during the daytime (Fig. 5B). Locomotor function at night was nominally, but not significantly, increased in aged mice compared to daytime. The locomotor function of the aged DSW-treated mice at night was significantly higher than that of the age-matched control. The daytime locomotor function of the young DSW-treated mice was significantly lower than that of the aged DSW-treated mice. Fig. 5C shows the locomotion results obtained for each mouse. Comparison of the groups indicated that young mice were more active at night, whereas aged mice were more active over the whole day.

### 3.5 Effect of DSW treatment on respiration in mice during rotation cage running

**Fig. 6:**
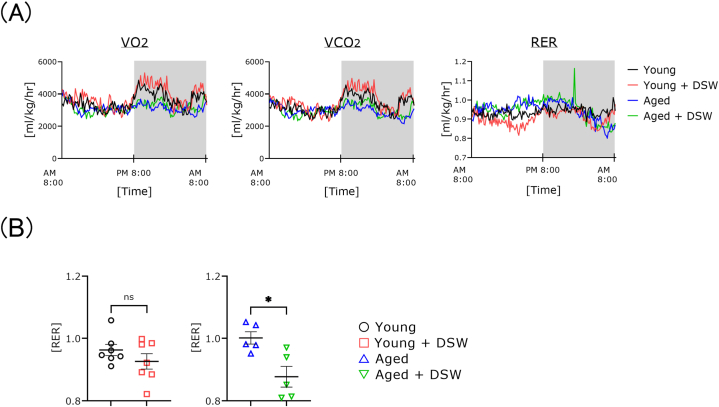
Effects of DSW treatment on changes in metabolic capacity and RER values in young and aged mice during rotation cage running. RER values were calculated by dividing the VCO_2_ value by the VO_2_ value, which were obtained concurrently (A). Mice were placed individually in the respiratory quotient measuring device, and respiratory metabolic capacity was measured for 24 h after 2 days of acclimatization. VO_2_ and VCO_2_ were automatically recorded for 10 min each. The groups consisted of Young (n = 6), Young + DSW (n = 6), Aged (n = 6), and Aged + DSW (n = 6). Data are shown as means ± SE. Data were analyzed using a two-way analysis of variance (A) and an unpaired *t*-test (B). No significant differences were observed (A). Changes in RER value when running in the rotation cage in DSW-treated young and aged mice (B). Rotation speed (m/min) and time were recorded automatically every 10 min. The groups consisted of Young (n = 7), Young + DSW (n = 7), Aged (n = 5), and Aged + DSW (n = 5). Young and Young + DSW were subjected to 10 m/min running. Aged and Aged + DSW were subjected to 0.2 m/min running. ∗*p* < 0.05.

VO_2_ and VCO_2_ scores were nominally higher, albeit not significantly), in young mice treated with DSW compared to the untreated age-matched controls. This tendency was most apparent during the night (Fig. 6A). To compare the metabolic capacity, RER scores at rotational speeds of 10 m/min in young mice and 0.2 m/min in aged mice were selected. The RER scores were not significantly different between young animals with or without DSW treatment. However, the scores of aged mice were significantly decreased in the DSW group compared to the untreated one (Fig. 6B).

### 3.6 Western blotting

Multiple bands were detected for BDNF and TrkB. There were no significant differences in all samples.

### 3.7 Changes in serum parameters of DSW-treated young and aged mice

Albumin was significantly decreased in aged mice with and without DSW treatment compared to the young mice (Fig. 8). Amylase and total cholesterol of aged mice were significantly increased compared to the young mice. Calcium and aspartate aminotransferase were significantly increased in the DSW-untreated aged mice compared to the young DSW-untreated mice. Creatinine, and sodium levels of aged mice with DSW treatment were significantly increased compared to the young DSW-treated mice.

## 4 Discussion

### 4.1 DSW does not affect body weight in young and aged mice

Minerals or inorganic ions play very important roles in the human body. For example, iron is bound to hemoglobin in erythrocytes [20], and magnesium is involved in muscle contraction and nerve transmission [21]. Co-treatment of selenium and vitamin E has been shown to significantly mitigate the effects of Alzheimer's disease [22]. Zinc and manganese each play a role as the catalytic center of the enzyme superoxide dismutase [23]. Calcium is important in intracellular signaling pathways and disruption of calcium homeostasis induces severe cellular dysfunction [24].

Large amounts of mineral water are sold commercially, and although the market is expanding annually [25], the water used in these products is typically collected in mountains and the main mineral component is calcium, with trace amounts of other minerals [26]. In contrast, the primary mineral component of DSW is magnesium, and numerous other minerals are present. Analyses using inductively coupled plasma atomic emission spectroscopy and inductively coupled plasma mass spectrometry have detected more than 20 elements in DSW [14], indicating that there is clear difference between the two types of water. Terrestrial animals evolved from marine animals millions of years ago, and the composition of their extracellular fluid is similar to that of seawater, with high amounts of sodium and chloride. Consequently, the amounts of minerals contained in DSW may be beneficial for our cellular physiologies. Our current research hypothesis is based on this idea.

In the present study, water with a hardness of 300 mg/L did not affect the body weight of young and aged mice, and water intake did not differ significantly among all groups (Fig. 2). There was no change in food intake with or without DSW treatment, and no diarrhea symptoms were observed. Although we detected a slight unique taste when drinking the DSW ourselves, based on the above facts, we concluded that there would be no issues with using this water for the experimental animals.

### 4.2 DSW enhances training effects in young and aged mice

Running distance decreased nominally after 2 months in all groups, albeit not significantly (Fig. 3). Animals were housed individually in small cages and were given access to food and water ad libitum. Since the space in which the mice could move was limited, it is possible that they lacked exercise and thus were only able to run a shorter distance. After 2 months of drinking each type of water, treadmill training was initiated (Fig. 4). During training, the mice consumed large quantities of oxygen and produced heat, which is thought to cause muscle fatigue. In the post-training endurance test, mice treated with DSW showed significantly higher training effects in both age groups. Our results corroborate the meta-analysis of Aragón-Vela et al. [27], who showed that drinking DSW significantly improved the ability of humans to recover muscle strength after exercise. One reason for the beneficial effects on treadmill training may be the magnesium in the water. The DSW contains very high amounts of magnesium and has a magnesium to calcium ratio of 3:1; conversely, most mineral water is high in calcium [14]. The relationship between muscle fatigue and magnesium is well-documented. For example, in a double-blind study in humans, treatment with magnesium (300 mg/day for 10 days) reduced muscle pain and improved performance [28]. Barbagallo et al. [29] reported that magnesium deficiency is associated with increased cardiovascular events and oxidative stress production. Further, magnesium deficiency has been implicated in muscle damage and exacerbating inflammation [27]. During exercise, heat is produced and inflammation occurs locally in the muscles, causing macrophages to accumulate. Macrophages are activated leading to excessive release of pro-inflammatory cytokines and free radical production [30]. Several minerals, including magnesium, are also closely related to enzymatic reactions and protein kinase activity [31]. For this reason, DSW treatment may have had a positive influence on the training effect. However, we did not measure fatigue indices such as lactic acid in the blood immediately after training; thus, further investigation is needed to substantiate this hypothesis.

### 4.3 DSW does not improve circadian rhythms but enhances motor performance in aged mice

To clarify the mechanism of the effect of DSW treatment on the exercise training effect in young and aged mice, we measured the respiratory quotient using a respiratory metabolism measuring device (Fig. 5). Several papers have reported that circadian rhythm change during aging [32]. In addition, many of the diseases that show increased risk with aging, such as Alzheimer's disease and type 2 diabetes, are closely related to circadian rhythm dysregulation [33]. Our aged mice were over 90 weeks of age during the metabolic cage experiments. In this context, the circadian rhythm of the aged mice may have been disturbed. DSW treatment did not improve circadian rhythms in aged mice, and DSW-treated young mice did not show increased locomotor function during the night. However, in aged mice, DSW treatment significantly increased locomotor function at night compared to the untreated group. These results showed that DSW treatment enhances locomotor function in aged mice. Previously, we assessed locomotor function in young high-fat diet-treated mice with the same DSW using a Rota-rod. The score for “time-to-fall” in the treated group was better than that of the untreated obesity group [14]. Taken together, while DSW treatment does not appear to modify circadian rhythms, it may increase locomotor function. While the reason for this is currently unclear, one possibility is that certain minerals have stimulatory or alertness-promoting effects. There are also reports on minerals and autonomic nerve stimulation, but the mechanistic details are sparse [34]. DSW may also have an effect on the intestinal flora. Further investigation is needed to clarify the mechanism of locomotor ability function and trace elements.

VO_2_ and VCO_2_ results were nominally increased in the young DSW-treated rats early in the night compared to their age-matched controls (Fig. 6). On the other hand, the daytime RER of the young DSW-treated rats was nominally lower than that of the age-matched controls. In the aged group, the values of VO_2_, VCO_2_ and RER were unaffected by the presence or absence of DSW treatment. The RER scores during the same rotation speed in the young DSW-treated rats tended to be lower compared to the untreated controls. However, the RER score in aged mice at the same rotation speed were significantly lower in DSW-treated mice compared to the untreated group. Hou et al. [26] reported that DSW supplementation had a significant effect on aerobic recovery. Numerous reports have been published on the relationship between physical activity and minerals [35]. Magnesium deficiency impairs physical performance, and the intake of magnesium salts improves cell function and increases muscle strength [36]. Iron is closely related to oxygen transport, and physical training distorts iron homeostasis [37]. Maynar et al. [38] reported on the importance of serum levels of trace elements and athletic performance. These results indicate that DSW treatment may increase metabolic capacity during nocturnal locomotor exercise in young mice and during exercise in the aged group. In addition, these results suggest that magnesium, which is relatively abundant in DSW, may also have a marked effect on wide range of motor functions. However, it remains to be clarified whether individual elements or a mixture of elements are responsible for the beneficial effects of DSW. This question is very important for future research.

### 4.4 DSW does not affect brain neurotrophic factor secretions in young and aged mice

**Fig. 7:**
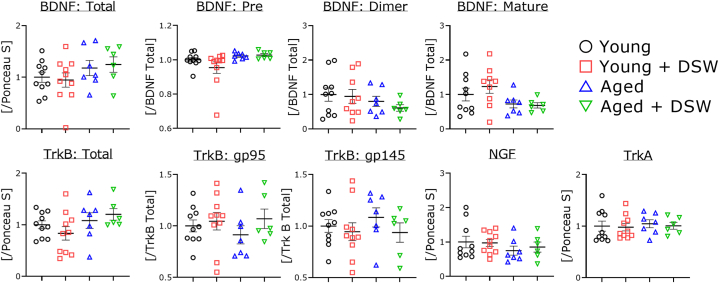
Western blot analysis of neurotrophic factor-related proteins in the brains of young and aged mice with and without DSW treatment. All experiments were performed using tissues from the cortex region. The ratio of each protein band intensity (BDNF total, TrkB, NGF, and TrkA) to Ponceau S intensity is shown, with ratios of control samples set to 1. The ratios of other protein intensities to each total protein intensity are shown, with ratios of control samples set to 1. The groups of consisted of Young (n = 10), Young + DSW (n = 10), Aged (n = 7), and Aged + DSW (*n* = 6). Data are shown as means ± SE. Comparisons were performed using the Tukey-Kramer method and there were no significant differences between samples.

**Fig. 8:**
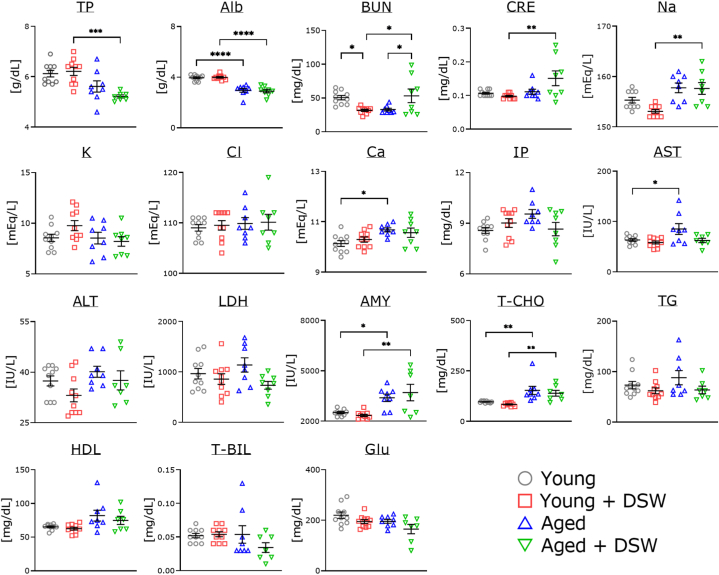
Changes in serum parameters of young and aged mice with and without DSW treatment. The groups consisted of Young (n = 10), Young + DSW (n = 10), Aged (n = 8), and Aged + DSW (n = 7 or 8). The data are shown as means ± SE. Comparisons were performed using the Tukey-Kramer method. ∗*p* < 0.05, ∗∗*p* < 0.01, ∗∗∗*p* < 0.001, ∗∗∗∗*p* < 0.0001. TP, total protein; Alb, albumin; BUN, blood urea nitrogen; CRE, creatinine; Na, sodium; K, potassium; Cl, chlorine; Ca, calcium; IP, inorganic phosphorus; AST, aspartate aminotransferase; ALT, alanine aminotransferase; LDH, lactic acid dehydrogenase; AMY, amylase; T-CHO, total cholesterol; TG, triglyceride; HDL, high density lipoprotein; T-BIL, total bile acid; Glu, glucose.

Exercise stimulates the brain and promotes blood flow [39] and neurostimulation through the release of neurotrophic factors may be effective in preventing dementia and activating brain function [40]. Erickson et al. [41] reported that exercise increases hippocampal size and prevents cognitive decline. Before starting the Western blot analysis, we expected that the expressions of BDNF and NGF, or their receptors, would be increased after DSW treatment in all groups. However, the findings showed that were no significant differences in any indices (Fig. 7), implying that minerals may affect motor and muscle functions to a greater extent than brain function. Of the serum indices, BUN increased significantly in the aged DSW-treated mice compared to the age-matched controls (Fig. 8). The reasons for this are unknown. However, these same indices did not increase in the young DSW-treated mice compared to the young untreated group. Sodium levels tended to be low in the young DSW-treated mice compared to the age-matched untreated mice, and a similar result was observed for pancreas (AMY), kidney (BUN and CRE), and liver indexes (ALT). These results indicate that DSW administration does not adversely affect the function of various organs in young mice. However, aged mice might show effects from DSW treatment. It is possible that the mice may have been too old or that the water hardness too high. These issues will be clarified in greater detail in future studies.

## 5 Conclusions

In our study, DSW treatment enhanced the exercise training effect in young and aged mice via changes in metabolic rate. The locomotor ability of aged mice was significantly increased with DSW treatment. The trace elements responsible for these effects are unclear. Furthermore, mitochondria, adenosine triphosphate and myoglobin levels should be measured to clarify the effects of DSW on training. Although we were unable to address these points at this time, these aspects could potentially be studied in future. DSW intake has been reported to reduce the risk of atherosclerosis, diabetes and obesity in human and animal models [42]. Presently, people of all ages suffer from mineral deficiency [3,4]. Thus, the general public as well as athletes could benefit from the continuous intake of DSW to mitigate the impacts of lack of exercise and sarcopenia-associated frailty, as well as general health maintenance.

## CRediT authorship contribution statement

**Koji Fukui:** Writing – review & editing, Writing – original draft, Visualization, Validation, Supervision, Software, Project administration, Methodology, Investigation, Funding acquisition, Formal analysis, Data curation, Conceptualization. **Riki Takeuchi:** Data curation. **Yugo Kato:** Investigation, Data curation. **Nozomu Takeuchi:** Resources. **Hirotsugu Takenaka:** Resources. **Masahiro Kohno:** Project administration, Investigation, Conceptualization.

## Data availability statement

All experimental data can be found in this paper and previously published papers (Reference No. 12). The raw data used and analyzed as part of the study are available from the corresponding author on reasonable request.

## 6 Animal ethics

The experiment was approved by the Animal Protection and Ethics Committee of Shibaura Institute of Technology (Approval No. #20003, approved on August 25, 2020).

## Declaration of competing interest

The authors declare that they have no known competing financial interests or personal relationships that could have appeared to influence the work reported in this paper.
